# Development of tooth regenerative medicine strategies by controlling the number of teeth using targeted molecular therapy

**DOI:** 10.1186/s41232-020-00130-x

**Published:** 2020-09-01

**Authors:** Katsu Takahashi, Honoka Kiso, Akiko Murashima-Suginami, Yoshihito Tokita, Manabu Sugai, Yasuhiko Tabata, Kazuhisa Bessho

**Affiliations:** 1 Department of Oral and Maxillofacial Surgery, Graduate School of Medicine, Kyoto University, Shogoin-Kawahara-cho 54, Sakyo-ku, Kyoto, 606-8507 Japan; 2 Department of Perinatology, Institute for Developmental Research, Aichi Human Service Center, Kasugai, Aichi Japan; 3 Department of Molecular Genetics, Division of Medicine, Faculty of Medical Sciences, University of Fukui, Fukui, Japan; 4 Department of Biomaterials, Institute for Frontier Medical Sciences, Kyoto University, Kyoto, Japan

**Keywords:** Tooth regeneration, Molecular targeted therapy, Usag-1, Cebpb, Supernumerary teeth, Third dentition, SOX2, Odontogenic epithelial stem cells

## Abstract

Analysis of various genetically modified mice, with supernumerary teeth, has revealed the following two intrinsic molecular mechanisms that increase the number of teeth. One plausible explanation for supernumerary tooth formation is the rescue of tooth rudiments. Topical application of candidate molecules could lead to whole tooth formation under suitable conditions. Congenital tooth agenesis is caused by the cessation of tooth development due to the deletion of the causative gene and suppression of its function. The arrest of tooth development in *Runx2* knockout mice, a mouse model of congenital tooth agenesis, is rescued in double knockout mice of *Runx2* and *Usag-1.* The *Usag-1* knockout mouse is a supernumerary model mouse. Targeted molecular therapy could be used to generate teeth in patients with congenital tooth agenesis by stimulating arrested tooth germs. The third dentition begins to develop when the second successional lamina is formed from the developing permanent tooth in humans and usually regresses apoptotically. Targeted molecular therapy, therefore, seems to be a suitable approach in whole-tooth regeneration by the stimulation of the third dentition. A second mechanism of supernumerary teeth formation involves the contribution of odontogenic epithelial stem cells in adults. Cebpb has been shown to be involved in maintaining the stemness of odontogenic epithelial stem cells and suppressing epithelial-mesenchymal transition. Odontogenic epithelial stem cells are differentiated from one of the tissue stem cells, enamel epithelial stem cells, and odontogenic mesenchymal cells are formed from odontogenic epithelial cells by epithelial-mesenchymal transition. Both odontogenic epithelial cells and odontogenic mesenchymal cells required to form teeth from enamel epithelial stem cells were directly induced to form excess teeth in adults. An approach for the development of targeted therapeutics has been the local application of monoclonal neutralizing antibody/siRNA with cationic gelatin for USAG-1 or small molecule for Cebpb.

## Background

The development of preemptive medicine, to extend healthy life expectancy in a super-aging society, is an important aspect of Japan’s medical strategy. “Eating” to improve oral frailty has been adopted, and the importance of dental treatment has been highlighted. Many instances of missing teeth can be attributed to acquire causes such as dental caries and periodontal disease, whereas congenital causes include congenital edentulous disease with a high incidence at 1% [1].

The basic treatment for missing teeth is prosthetic replacement with dental implants or dentures. These prosthetic treatments have been traditionally used and are still being used and further developed at present. Preemptive medicine for tooth regeneration is expected to become incorporated into clinical practice. Studies of tooth regeneration using tissue engineering approaches have been reported. Various cells such as stem cells are used as a cell source. In addition, to make teeth in vitro [2], the “organ germ method” has been reported, which is a cell manipulation technology that regenerates the organ primordium of the tooth in a collagen gel [3]. However, all current tissue engineering approaches have problems, such as the cost and safety of the cell source and concerns regarding the potential for contamination or tumorigenicity, and thus, they have not yet reached readiness for clinical applications. Further to this, antibody drugs, which are molecularly targeted drugs, are being developed not only for cancer but also for various other diseases. Patisiliane, an siRNA, is currently being investigated as a drug for familial amyloidosis [4]. By combining genomic analysis, epidemiological research, and mouse model studies, we have consistently aimed to regenerate teeth by controlling the number of teeth using molecular approaches, from gene therapy with viral vectors, to targeted molecular therapy with antibody- or siRNA-based drugs.

Humans are diphyodonts and have both residual and permanent teeth, including incisors and premolars. Rodents, such as mice, have a retarded number of teeth, with only one incisor and three molars, and as monophyodonts they have no residual teeth. Similar to many other organs, the number of teeth is strictly controlled within each species [5]. Analysis of various knockout mice with supernumerary teeth has revealed the following two intrinsic molecular mechanisms that increase the number of teeth [6–16]: (1) rescue of rudimental tooth germs and (2) contribution of odontogenic epithelial stem cells (OESCs).

In this article, we will introduce recent advances in research regarding these two mechanisms and discuss the potential of tooth regeneration with targeted molecular therapy, alongside descriptions of target molecules.

## Main text

### Rescue of rudimental tooth germs

Mice, unlike humans, have one incisor and three molars that are separated by a tooth formation-free region called the diastema. Several mechanisms have been proposed to account for the formation of supernumerary teeth in mice [14, 16, 17]. One plausible explanation for supernumerary tooth formation is the rescue of tooth rudiments in the diastema or maxillary deciduous incisors [14, 18–20]. Most reported mouse supernumerary teeth are located in the diastema region. This is the rescue of vestigial tooth rudiments. During the early stages of tooth development, many transient vestigial dental buds develop in the diastema area. Some of them can develop into the bud stage, but later regress and disappear by apoptosis, or merge with the mesial crown of the first molar tooth [21–26]. Major signaling pathways regulating tooth development are also expressed in these vestigial dental buds. A number of mutant mouse strains have been reported to exhibit supernumerary diastema teeth. Although rudimentary tooth buds form in the embryonic diastema, they regress apoptotically [27]. Transgenic mice in which the keratin 14 promoter directs ectodysplasin (Eda) or Eda receptor expression to the epithelium had supernumerary teeth mesial to the first molar as a result of diastema tooth development [28–30]. It is also reported that Sprouty2 (Spry2)- or Spry4-deficient mice develop supernumerary teeth as a result of diastema tooth development [31]. Usag-1 is a bone morphogenetic protein (BMP) antagonist [32]. We have previously demonstrated that inhibition of apoptosis can lead to successive development of rudimentary maxillary incisors in *Usag-1* null mice [16]. Furthermore, increased BMP signaling is observed in *Usag-1*-deficient mice, which prevents apoptosis and leads to the development of supernumerary teeth [14]. These results suggest that inactivation or inhibition of single candidate molecules such as Usag-1 has the potential to regenerate whole teeth through the rescue of the rudimental tooth germ. Furthermore, we have previously claimed that gene interactions between BMP-7 and Usag-1 regulate supernumerary maxillary incisor formation [12]. BMP-7 co-localizes with Usag-1 in the area of the maxillary rudiment incisor tooth germ, as well as the regular maxillary incisor tooth organ. We previously confirmed that increased BMP signaling in supernumerary teeth of Usag-1 deficient mice could be prohibited by BMP-7 abrogation. Using subrenal capsule transplantation of embryonic day 15 (E15) maxillary incisor tooth primordia supplemented to BMP-7 with cationic gelatin demonstrated rescue of tooth rudiments and supernumerary tooth development in both *Usag-1*^*+/−*^ and *Usag-1*^*−/−*^ mic*e*. Based upon these results, we claimed that Usag-1 functions as an antagonist of BMP-7 and that topical application of the candidate molecule could make a whole tooth under the correct conditions [12].

Many genes responsible for congenital tooth agenesis have been identified, and many are common in humans and mice [33]. Mouse model studies have demonstrated that congenital tooth agenesis is caused by the cessation of tooth development halfway through the process due to the deletion of the causative gene and suppression of its function. For example, RUNX2, MSX1, EDA, WNT10A, PAX9, and AXIN2 are known to be responsible for congenital tooth agenesis [34–37]. However, the majority of patients with congenital edentulous disease have causative mutations in WNT10A [38, 39]. EDA is a causative gene of anhidrotic ectodermal dysplasia, which is a representative disease of syndromic congenital edentulous disease [40]. *Runx2*^*−/−*^ mice exhibit stunted tooth formation [37], and patients with a unique Arg131Cys missense RUNX2 mutation develop a novel dental phenotype; i.e., they have no supernumerary teeth but one congenitally missing tooth [41]. In double null *Usag-1*^*−/−*^/*Runx2*^*−/−*^ mice, three interesting phenomena were observed: the prevalence of supernumerary teeth was lower than in *Usag-1* null mice; tooth development progressed further compared to *Runx2* null mice; and the frequency of molar lingual buds was lower than in *Runx2* null mice [9]. Therefore, we suggest that Runx2 and Usag-1 act in an antagonistic manner [9]. We previously demonstrated that deletion of *Usag-1* rescued the hypoplastic and poorly differentiated molar and incisor phenotypes observed in *Runx2*^*−/−*^ mice [9]. The rescue of tooth formation in genetically defined mouse models clearly demonstrates the feasibility of inducing de novo tooth formation via the in situ repression of a single targeted gene. Our investigations and related studies clearly validate the hypothesis that de novo repression of target genes, such as *Usag-1*, can be used to stimulate arrested tooth germs in order to induce new tooth formation in mammals. Conversely, it was reported that *Usag-1* abrogation could rescue cleft palate development but not tooth development arrest in Pax9 deficient mice. In the rescued palate phenotype, modulated WNT signaling was observed but no BMP signaling [42]. Small-molecule Wnt agonists also corrected cleft palate in Pax9 deficient mice [43]. However, it is currently unclear whether repression of Usag-1 could universally rescue the effects of other causative genes of congenital tooth agenesis. The rescue of tooth formation in genetically defined mouse models clearly demonstrates the feasibility of inducing de novo tooth formation via the in situ repression of a single targeted gene. Our investigations and related studies clearly validate the hypothesis that de novo repression of target genes, such as Usag-1, could be used to stimulate arrested tooth germs in order to induce new tooth formation in mammals. Indeed, in animal models of EDA deficiency, which is associated with the human disorder hypohidrotic ectodermal dysplasia which involves hypodontia, the administration of a soluble EDA receptor agonist has been shown to correct many phenotypic abnormalities, including abnormalities of the dentition in mice (primary) and dogs (secondary or permanent) [44–47].

**Fig. 1:**
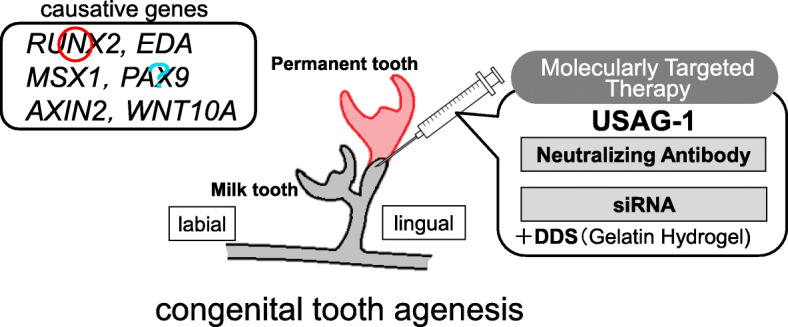
Treatment plan for congenital tooth agenesis. RUNX2, EDA, MSX1, PAX9, AXIN2, and WNT10A have been identified as causative genes for congenital tooth agenesis. The mutation of the causative gene is used as a biomarker, and a neutralizing antibody of USAG-1 or USAG-1 siRNA is administered as a molecularly targeted drug

After a single postnatal systemic administration of recombinant EDA or anti-EDA receptor agonist monoclonal antibody, it was confirmed that all the physiological aspects of missing teeth, such as the number, size, shape, location, and timing of eruption, were restored, and the teeth functioned normally [44, 47]. In fact, lifelong phenotypic correction was achieved after a short course of treatment [44, 47]. In the future, molecular targeted therapies could be used to generate teeth in patients with congenital tooth agenesis by stimulating arrested tooth germs (Fig. 1).

**Fig. 2:**
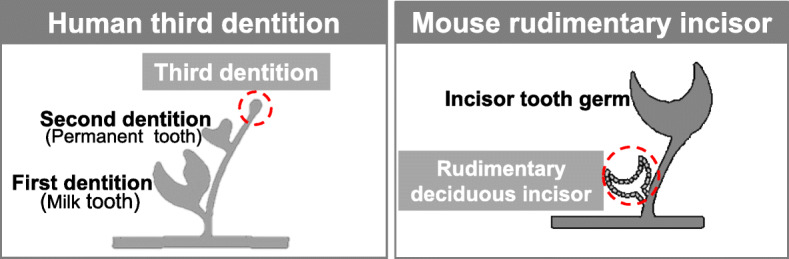
Expanding the target from congenital tooth agenesis to general missing teeth (the third dentition). Both the human third dentition and mouse rudimentary incisor usually regressed

**Fig. 3:**
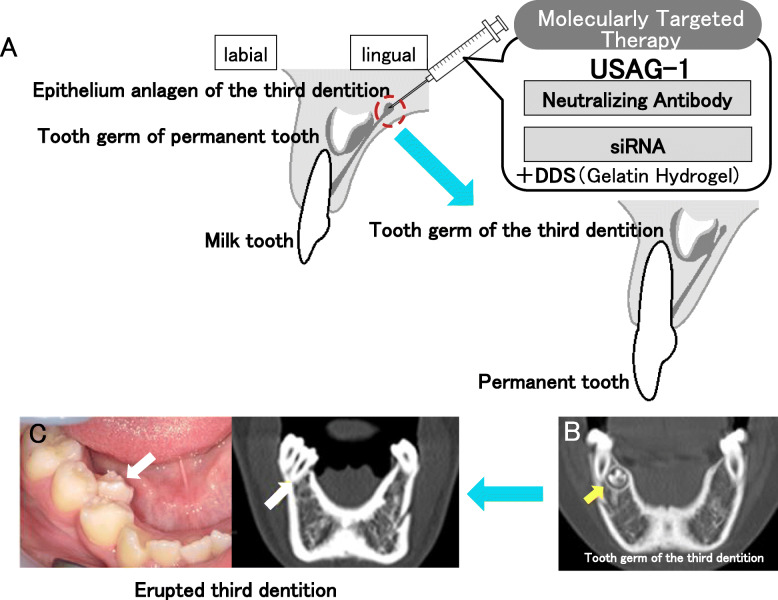
Tooth regeneration by stimulation of the third dentition. **a** This treatment involves regenerating the third dentition following permanent teeth eruption by locally administering a neutralizing antibody or siRNA with DDS like cationic gelatin hydrogel for USAG-1. **b** Frontal section of a computerized tomography (CT) scan of the tooth germ of the third dentition located in the lingual side of the lower right premolar that is preceding the permanent molar in an 11-year-old individual. The yellow arrow indicates the tooth germ of the third dentition. **c** Occlusal view and frontal section of a CT scan of the erupted third dentition in an 18-year-old individual. The white arrows indicate the erupted third dentition

The mechanisms underlying human supernumerary tooth formation have recently become clearer. Deciduous teeth are, ontogenetically, the first generation of teeth. The permanent teeth (except molars) belong to the second dentition. The term “third dentition” refers to the opinion that one more set of teeth can occur in addition to the permanent teeth [48, 49]. The third dentition begins to develop when the second successional lamina is formed from the developing permanent tooth in humans and usually regresses apoptotically like the rudimental incisor of the mouse (Fig. 2). It typically does not completely form the tooth structure [48, 49]. Recently, it was suggested that supernumerary teeth result from the rescue of the third dentition’s regression in humans [49–51]. Radiographic examination of tooth development in patients with cleidocranial dysplasia performed over several years suggests that a part of the third dentition may cause the condition [52]. Clinical criteria for supernumerary teeth derived from the third dentition are as follows: (1) the supernumerary tooth is located on the lingual side of permanent teeth, (2) the supernumerary tooth develops after permanent teeth formation, and (3) the shape of the supernumerary tooth is similar to that of the preceding teeth [53, 54]. We have previously reported that this clinical definition would apply to at least one-third of non-syndromic multiple supernumerary teeth [54]. Recently, our investigation evaluated the proportion of collected general supernumerary teeth cases that evinced a third dentition based on the clinical definition of supernumerary teeth derived from the third dentition [55]. The frequency of supernumerary teeth considered to have been derived from the third dentition was 26 out of 78 cases [55]. Evidence of a third dentition was especially apparent in the premolar region, was more common in men, and more likely to occur in patients with three or more supernumerary teeth [55]. It was suggested that the third dentition is the main cause of supernumerary teeth in humans. Our investigation demonstrated that the third dentition, possibly underlain by genetic factors, is a major cause of supernumerary teeth in humans—especially multiple supernumerary teeth [55]. The timing of appearance of the third dentition appears to be after birth [48], meaning that we have a chance to access its formation directly in the mouth. The duration of tooth regeneration by stimulation of the third dentition is almost the same as that of permanent tooth development, according to the clinical case of tooth regeneration by stimulation of the third dentition (Fig. 3). The third dentition has the potential to erupt following permanent teeth eruption without dental caries and periodontal disease (Fig. 3). We can utilize the healthy newly formed teeth for usual dental treatments, such as tooth extraction, orthodontic treatment, and tooth transplantation. Therefore, molecularly targeted therapy seems to be a suitable approach in whole-tooth regeneration by stimulation of the third dentition (Fig. 3).

### Contribution of OESCs

A second mechanism of ST formation involves the contribution of OESCs, whereas multiple other mechanisms based on mouse models have been developed [49, 50]. Sox2 is a molecular marker of OESs in mice [56], and Sox2-positive OESCs reportedly contribute to supernumerary tooth formation in mice [6, 57]. We previously demonstrated that Cebpb deficiency is related to the formation of supernumerary teeth. A total of 66.7% of *Cebpb*^*−/−*^ 12-month-old animals presented supernumerary teeth and/or odontomas [13]. There were significantly fewer Sox2-positive cells in the labial cervical loop epithelium of adult *Cebpb*^*−/−*^ mouse incisors than in wild-type animals [6]. These findings suggest that Cebpb maintains Sox2-positive OESCs in the labial cervical loop epithelium during postnatal life. Differentiated ameloblasts in the maxillary incisor were deranged and lost their cell polarity in adult *Cebpb*^*+/+*^*/Runx2*^+/*−*^ animals [6]. Furthermore, the disappearance of the apical-basal polarity of differentiated ameloblasts was visible in *Cebpb*^*−/−*^/*Runx2*^*+/+*^ and *Cebpb*^*−/−*^/*Runx2*^*+/−*^ mice, consistent with the epithelial-mesenchymal transition (EMT) process [58]. Thus, based on these morphological changes, we suggest that *Cebpb* and *Runx2* knockdown contributes to EMT in odontogenic epithelial cells of the maxillary incisor. Taken together, supernumerary tooth formation around the labial cervical loop epithelium of adult maxillary incisors may be dependent on both *Cebpb* knockdown-induced loss of stemness in OESCs and EMT of odontogenic epithelial cells in *Runx2*^+/*−*^ and/or *Cebpb*^*−*/*−*^ mice [6]. In Runx2 heterozygous and null mice, budding was observed in maxillary incisors at E15. Both Runx2 mutants displayed lingual buds in front of the maxillary molars, which are in line with Runx2 preventing the formation of buds for successional teeth [59, 60]. There is a difference between the phenotypes of OESCs in *Cebpb*^*−/−*^/*Runx2*^*+/+*^ and *Cebpb*^*−/−*^/*Runx2*^*+/−*^ incisors between E15 and adult animals. No buddings were observed at E15 in *Cebpb*^*−/−*^ mice but were seen at E15 in *Cebpb*^*+/+*^/*Runx2*^*+/−*^ and *Cebpb*^*−/−*^/*Runx2*^*+/−*^ mice, before disappearing on postnatal day 7 [6]. Meanwhile, some adult *Cebpb*^*−*/*−*^ mice possess an unusual incisor that presented ectopic hyperplasia of enamel and dentin in the periapical tissue. Moreover, 33% of 3-month-old *Cebpb*^*−*/*−*^/*Runx2*^+/*−*^ mice had aberrant incisors, characterized by developing or mature ectopic supernumerary teeth in the periapical tissue and dental pulp [8]. Indeed, in humans, supernumerary teeth are less common in the deciduous dentition (first generation of teeth) than in the permanent dentition (second generation of teeth) [61]. In mice, the difference may be linked to stem cell aging in the incisor. Common contributing factors of aging in different organisms, but particularly in mammals, are genomic instability, telomere attrition, epigenetic alterations, loss of proteostasis, deregulated nutrient sensing, mitochondrial dysfunction, cellular senescence, stem cell exhaustion, and altered intercellular communication [62]. As another example of epithelial-mesenchymal interactions, hair graying is the most obvious sign of aging in mammals. Irreparable DNA damage, as that caused by ionizing radiation, abolishes the renewal of mesenchymal stem cells (MSCs) in mice and results in hair graying inasmuch as it also triggers MSC differentiation into mature melanocytes in the niche [63]. The hallmarks of OESCs can change according to aging.

Studies show that conditional knockout of the Apc-gene results in the development of supernumerary teeth in mice [64–66]. Notably, adult oral tissues, particularly in young adults, are still responsive to loss of Apc [17]. In old adult mice, supernumerary teeth can be induced on both the labial and lingual sides of the incisors, which contain adult stem cells supporting the continuous growth of mouse incisors [17, 66]. In young mice, supernumerary tooth germs were induced in multiple regions of the jaw, in both incisor and molar regions. They could form directly from the oral epithelium, in the dental lamina connecting the developing molar or incisor tooth germ to the oral epithelium, in the crown region, as well as in the elongating and furcation area of the developing root [67]. Canonical Wnt/β-catenin signaling and its downstream molecule Lef-1 are essential for tooth development [68]. Overexpression of Lef-1 under control of the K14 promoter in transgenic mice develops abnormal invaginations of the dental epithelium in the mesenchyme and forms tooth-like structures [69]. De novo supernumerary teeth arising directly from the primary tooth germ or dental lamina have been reported in Apc loss-of-function or β-catenin gain-of-function mice. It was demonstrated that mouse tooth buds expressing stabilized β-catenin give rise to extra tooth formation [65].

**Fig. 4:**
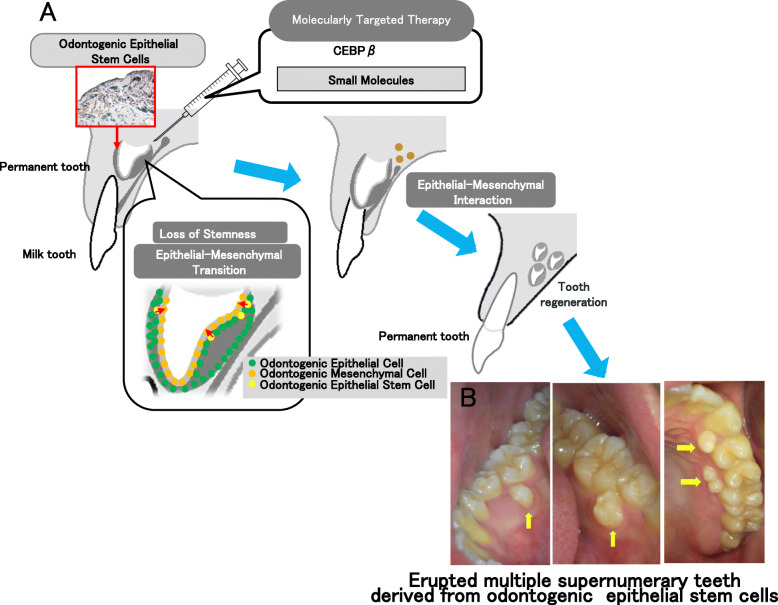
Development of tooth regenerative medicine targeting odontogenic epithelial stem cells. **a** Cebpb is involved in maintaining the stemness of enamel epithelial stem cells and suppressing epithelial-mesenchymal transition (EMT). Odontogenic epithelial stem cells (OESCs) are differentiated from one of the tissue stem cells; enamel epithelial stem cells and odontogenic mesenchymal cells are formed from odontogenic epithelial cells by EMT. It was demonstrated that both odontogenic epithelial cells and odontogenic mesenchymal cells, required to form teeth from enamel epithelial stem cells, were directly induced to form excess teeth. **b** Occlusal view of erupted multiple supernumerary teeth derived from odontogenic epithelial stem cells. The yellow arrows indicate the erupted multiple supernumerary teeth

Furthermore, SOX2-positive OESCs are reportedly related to several different odontogenic diseases in humans [57, 70, 71]. We also investigated the association between supernumerary teeth and SOX2-positive OESCs and found that some instances of human supernumerary teeth, except those derived from the third dentition, were caused by Sox2-positive OESC, as was observed in Cebpb and Runx2 double knock-out mice [6]. However, it may be possible that OESCs contribute to supernumerary tooth formation in general. Tooth development is under genetic control and is the result of reciprocal and reiterative signaling between the oral ectoderm-derived dental epithelium and cranial neural crest cell-derived dental mesenchyme [72]. Two different cell types (epithelial and mesenchymal) are necessary and essential for whole-tooth regeneration. OESCs have the potential to differentiate odontoblasts by loss of stemness and EMT and regenerate de novo whole teeth (supernumerary teeth) by ectomesenchymal abrogation of *Cebpb* (Fig. 4). In recent years, there have been many studies of stem cells in relation to the development of regenerative therapies for various organs such as nephrons (the functional units of the kidney) or the pituitary gland [73, 74]. We also demonstrated that OESCs may contribute to supernumerary teeth formation in cases not caused by third dentitions [55]. Multiple supernumerary teeth derived from odontogenic epithelial stem cells have the potential to erupt following the eruption of permanent teeth without dental caries and periodontal disease (Fig. 4). We can utilize the healthy newly formed teeth for usual dental treatments. OESCs are suitable for molecular targeted therapy for whole-tooth regeneration using Cebpb (Fig. 4).

## Conclusions

One plausible explanation for supernumerary tooth formation is the rescue of tooth rudiments. Congenital tooth agenesis is caused by the cessation of tooth development due to the deletion of causative genes and suppression of their function. Molecular targeted therapy could be used to generate teeth in patients with congenital tooth agenesis by stimulating arrested tooth germs. The third dentition begins to develop when the second successional lamina is formed from the developing permanent tooth in humans. Molecularly targeted therapy therefore seems to be a suitable approach in whole-tooth regeneration by the stimulation of the third dentition. A second mechanism of supernumerary tooth formation involves the contribution of OESCs. Cebpb has been shown to be involved in maintaining the stemness of OESCs and suppressing epithelial-mesenchymal transition in adults. OESCs are differentiated from one of the tissue stem cells, enamel epithelial stem cells, and odontogenic mesenchymal cells are formed from odontogenic epithelial cells by EMT. A major approach for the development of targeted therapeutics has been the local application of monoclonal neutralizing antibodies or siRNAs with cationic gelatin for Usag-1 or small molecule for Cebpb. However, for future clinical applications, further safety studies investigating the toxicity, teratogenicity, and tumorigenicity of these therapeutic molecules need to be performed.

## Data Availability

Not applicable

## Abbreviations

EMT: Epithelial-mesenchymal transition
OESC: Odontogenic epithelial stem cell
E15: Embryonic day 15
MSC: Mesenchymal stem cell
